# Interactive Fluid Coupling Effects of Non-Neighbouring Members

**DOI:** 10.3390/s21216961

**Published:** 2021-10-20

**Authors:** Arun Kumar Manickavasagam, Stefanie Gutschmidt, Mathieu Sellier

**Affiliations:** Department of Mechanical Engineering, University of Canterbury, Christchurch 8041, New Zealand; stefanie.gutschmidt@canterbury.ac.nz (S.G.); mathieu.sellier@canterbury.ac.nz (M.S.)

**Keywords:** interactive fluid coupling, arrays, non-neighbouring members, sensors, biomedical devices, boundary integral technique

## Abstract

Broadband, multi-functional and parallel-processing devices are often built on coupled oscillators or arrays of resonators. Different length scales and applications determine the dominating coupling mechanism of the device. In this paper we investigate the effects of interactive fluid coupling between members of a one-dimensional array wherein only one member is actuated. We are specifically interested in studying the influence of non-neighbouring members in small-size arrays comprising of three and five members for different Reynolds numbers and gap widths between members. Our model and analysis is based on the Navier–Stokes equation for incompressible flow which is solved using a boundary integral technique resulting in the hydrodynamic coupling matrix through which added mass and damping effects are inferred. Results clearly suggest that non-neighbouring members play a significant role for most typical array configurations and therefore cannot be ignored. In particular, arrays with more than three members must account for the behaviour of such a device with all member interactions. Thus, predicting the performance of most new and emerging technologies such as sensors and biomedical devices is determined by array effects rather than local, nearest neighbour influences.

## 1. Introduction

There has been growing interest in understanding the collective dynamics of oscillators, especially in a fluid environment both at micro- and macro-scales. Some of the applications of interest are fluidic coupled oscillators [1], chemically coupled oscillators [2], MEMS (micro-electromechanical systems)-based technologies such as, e.g., scanning probe microscopy [3] and energy harvesters [4,5,6,7]. Large scale applications include arrays of wind farms [8] and an array of densely packed buildings [9].

Dominating coupling effects can be of different origin (fluid, mechanical, thermal, etc.) and magnitude. At the macro scale, several researchers explored the coupling dynamics between a pair of cantilevers analytically and experimentally [10,11,12]. It was shown by Intartaglia et al. [12] that the added mass effect is magnified for decreasing gaps and hydrodynamic damping decreases as the gap increases. Their analysis was performed over a range of Reynolds numbers, i.e., O(10) to O(1000). Their proposed theoretical approach was also validated experimentally in water on centimetre-sized compliant beams subject to base excitation. Cellini et al. [13] investigated hydrodynamic coupling effects in a parallel array (face-to-face configuration) of five identical ionic polymer metal composites (IPMCs) subjected to low frequency base excitation, limiting the interactions only to nearest neighbours. Their analysis suggests that closely spaced IPMCs result in higher harvested powers, which is also validated experimentally.

At the micro scale, Hosaka and Itao [14] and Clark and Paul [15] investigated the fluid-coupled vibration of two micro cantilevers theoretically and experimentally. Basak and Raman [16] studied the hydrodynamic coupling effects of an array of five members considering only nearest neighbour coupling. The array under investigation was an edge-to-edge configuration and the authors studied the effects of hydrodynamic forces for a range of gap widths, amplitude ratios and relative phases analytically and computationally. They concluded that the dynamics of members in an array can be tuned to either maximize or minimize the overall effective hydrodynamic loading on individual microbeams. Ghatkesar et al. [17] performed experiments on an array of eight microcantilever beams immersed in fluids and verified the experimental results of eigenfrequency with four available analytical models and found a good match with Sader’s extended viscous model [18]. While existing contributions have considered the nearest neighbour interactions in an array of oscillators, no existing work seems to include the effects of non-neighbouring members, e.g., [16,17]. Having worked in the field of MEMS arrays (in fluid or not) over the last decade and in particular, scanning probe microscopy, our curiosity drew us to the question of whether observed artifacts could be related to dynamic coupling effects of non-neighbouring members in an array.

While the hydrodynamic coupling effects of local neighbours in an array have been studied in detail by Basak and Raman [16], none of their existing investigations include non-neighbouring influences. Furthermore, we distinguish the use of terminology “fluid coupling” or “hydrodynamic coupling” in the literature to our definition of “interactive coupling”. For instance, when contributions investigated in the literature talk about added mass and damping effects, the meaning of “coupling” typically refers to an additional hydrodynamic load originating from fluidic effects on top of other mechanical, magnetic, gravitational loads on the system. To the author’s knowledge, there does not seem to exist an investigation of “coupling” in the sense of inter-member, relative coupling effects that manifest themselves locally as well as globally. Here, we define interactive coupling as the ratio of the hydrodynamic load between beams. It is important to understand such interactive coupling effects especially with respect to high-precision applications such as, e.g., lithography or scanning probe microscopy [3,19].

In this work, we focus purely on interactive coupling effects between members of the array and the ways in which these influence the local and collective dynamics of the array. More specifically, we investigate the hydrodynamic influences of non-neighbouring members in small-size arrays. The aim of this work is to be able to distinguish between parameter domains at which coupling effects of non-neighbouring members significantly influence or even determine the overall performance of the system and for which these can be neglected. We perform our analysis on small-size arrays with three and five beams in an edge-to-edge configuration considering motion in transverse direction.

The article is organized as follows: in Section 2 we present the general formulation of the array model resulting in a simplified equation for the streamfunction and respective velocity components in the transverse and lateral directions. In Section 3 we include a brief review of the models in the literature for hydrodynamic loading across a thin ribbon and a pair of beams which sets the foundation for an array analysis. In Section 4 hydrodynamic coupling effects of an array comprising of three and five members are investigated for different gap widths and Reynolds numbers. In Section 5 a distinction is drawn between local and global effects together with the gaps at which non-neighbouring members become significant. Moreover, some relevant applications that can exploit or avoid the effect of non-neighbouring members are discussed.

## 2. General Formulation of the Array Model

### 2.1. Model Description

**Figure 1:**
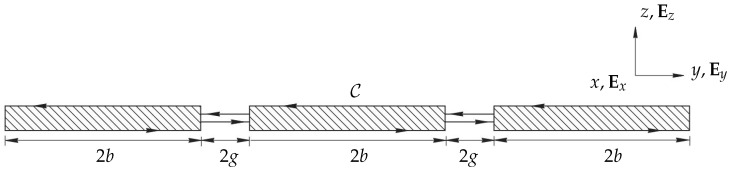
Sketch of the boundary value problem for three oscillating rectangular cross-sectional beams. $E_{x}$, $E_{y}$, $E_{z}$ is the vector basis corresponding to the *x*, *y* and *z* coordinate system.

Figure 1 depicts a schematic sketch of cross-sectional view of cantilevers in fluid in which the beams are long, slender cantilever structures immersed in an incompressible, viscous fluid. We consider two-dimensional profiles of cantilevers placed in an unbounded fluid domain, each of width 2b and spaced 2g apart. Structural coupling effects are ignored with only the motion of the fluid surrounding the profiles when one member is actuated being considered. The motion is considered in the z-direction only with its velocity component being ω. The fluid motion of this system can be modelled by the linearized Stokes and the continuity equation. The Fourier transformed unsteady Stokes and continuity equations for the fluid are given by [20]

(1) $$-i\omega \rho u=-\nabla P+\mu \nabla^{2}u,\;\nabla \cdot u=0,$$

where ω is the driving frequency, u(y,z|ω) is the fluid velocity vector given by $u=v\left(y,z|\omega \right)E_{y}+w\left(y,z|\omega \right)E_{z}$, where *v* and ω are magnitudes of velocities in the lateral and transverse directions, respectively, P(y,z|ω) is the pressure field in the fluid, and ρ and μ are the density and dynamic viscosity of the fluid, respectively. The beam cross sections in the y−z plane are separated from the fluid domain by a closed contour C.

We base the derivations of mathematical expressions on the following assumptions:

(1) Each beam has a rectangular cross section that remains uniform along its length.
(2) The fluid motion along the axial direction $E_{x}$ can be neglected for lower flexural modes.
(3) Only transverse vibrations of the beam along $E_{z}$ are considered and any lateral motion along $E_{y}$ is ignored.
(4) Only hydrodynamic coupling effects are considered, ignoring any effects arising from structural coupling.
(5) The fluid is incompressible as the acoustic wavelength in both liquids and gases typically exceeds the characteristic length scale of the beam.
(6) Only one beam in the array is considered to be actuated while the rest of the beams are passive in order to study the inter-member coupling effects.

### 2.2. Streamfunction Formulation

In this section, we implement the boundary integral method given by Pozrikidis [21] and extend the formulation given by Tuck [20] and Basak and Raman [16] for an array of *M* beams in general matrix form. The final expression determines the hydrodynamic forces along the width of each beam. We consider small amplitude oscillations of infinitely thin cantilever beams of a rectangular cross section, as shown in Figure 1. We, however, highlight new and additional terms arising due to non-neighbouring members and compare, validate and discuss our results against theirs.

The far-field boundary condition is that u→0 as y,z→±∞ and the velocity at the solid-fluid interface is given by [20]:

(2) $$v=0,\;w=W_{m},$$

at z = 0 (beam), where $W_{m}$ is the transverse velocity of the beam cross section for an arbitrary beam *m* with *m* being an integer between 1 and *M*.

Following (2), we now introduce a stream function ψ(y,z) to satisfy the continuity equation in (1)

(3) $$v=\psi_{z},\;w=-\psi_{y}.$$

Thus, the boundary conditions at the solid–fluid interface in terms of the streamfunction are

(4) $$\psi_{z}=0,\;-\psi_{y}=W_{m}.$$

Reformulating Equation (3) in terms of the streamfunction and application of Green’s theorem yields the following expression for the streamfunction; [16]:

(5) $$\begin{array}{c} \psi \left(y,z|\omega \right)=\int_{C}(\psi \left(y^{\prime },z^{\prime }|\omega \right)G_{n}\left(y,z|y^{\prime },z^{\prime }\right)-\psi_{n}\left(y^{\prime },z^{\prime }|\omega \right)\Omega \left(y,z,|y^{\prime },z^{\prime }\right)\\ \;\;\;\;\;\;\;\;\;\;\;\;\;\;\;\;\;\;\;\;\;\;\;\;\;\;\;\;\;\;\;\;\;\;\;\;\;\;\;\;\;\;\;\;\;-\zeta \left(y^{\prime },z^{\prime }|\omega \right)\Psi_{n}\left(y,z|y^{\prime },z\right)+\frac{1}{\mu }P\left(y^{\prime },z^{\prime }|\omega \right)\Psi_{l}\left(y,z|y^{\prime },z^{\prime }\right))dl, \end{array}$$

where (y,z) are the coordinates of a point in the fluid domain, $\left(y^{\prime },z^{\prime }\right)$ are the coordinates of a point on the contour C, ζ is the fluid vorticity, and G,Ω and Ψ are the Green’s functions for the Laplace operator, the Helmholtz operator and the operator $\nabla^{4}\left(.\right)-iRe\nabla^{2}\left(.\right)$, respectively. The subscripts *n* and *l* define derivatives in normal (transverse) and parallel (lateral) directions to the contour C, respectively.

The Green’s function Ψ is given by $\Psi =\frac{1}{\alpha^{2}}\left(G-\Omega \right)$[21], where $G=\frac{1}{2\pi }\log R$, $\Omega =\frac{-1}{2\pi }K_{0}\left(\alpha R\right)$, $\alpha =\sqrt{iRe}$, $K_{0}$ is the modified Bessel function of third kind, order zero and $R=\sqrt{{\left(y-y^{\prime }\right)}^{2}+{\left(z-z^{\prime }\right)}^{2}}$.

Assuming no relative motion between the top and bottom faces of the beam requires both Ψ and $\Psi_{n}$ to be continuous across the beam. As a consequence, the first two terms in the Equation (5) cancel out resulting in the following simplified equation [16]:

(6) $$\psi \left(y,z|\omega \right)=\int_{C}-\zeta \left(y^{\prime },z^{\prime }|\omega \right)\Psi_{n}\left(y,z|y^{\prime },z^{\prime }\right)+\frac{1}{\mu }P\left(y^{\prime },z^{\prime }|\omega \right)\Psi_{l}\left(y,z|y^{\prime },z^{\prime }\right))dl,$$

**Figure 2:**
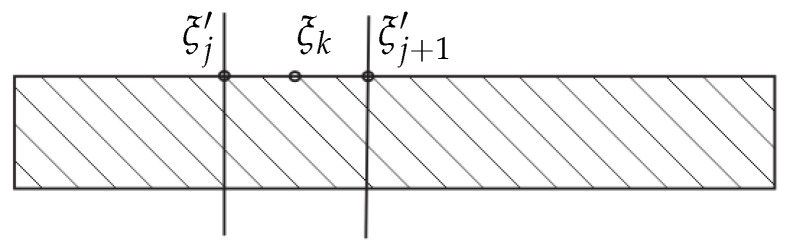
Cross-section showing a segment with nodes $\xi_{j}^{\prime }$ and $\xi_{j+1}^{\prime }$ and the midpoint $\xi_{k}$.

In order to obtain the velocity components, Equation (6) is differentiated with respect to *z* and *y* resulting in coupled integral equations. A numerical scheme is used to convert the system of integral equations into a corresponding system of matrix equations using the numerical quadrature method [22]. The beam is discretized into *N* segments. To avoid square root singularities, an unequal discretization method is employed. We evaluate the modified Equation (6) at the beam’s midpoints. The existence of logarithmic singularity makes the matrix compliant in its inversion properties and hence we do not eliminate it. Matrix entries are computed using Gauss–Legendre quadrature [22] to obtain the unknown pressure differences across the width of the beam which are approximated to be constant over each segment. One could also solve these equations for vorticity jumps when the beams move laterally. However, here we focus only on the transverse motion which is of primary interest contributing to the pressure profiles across the width of the beam. More details on the application of the boundary-integral method can be found in Pozrikidis [21]. The corresponding nondimensional hydrodynamic force per unit length acting on the beams is given by [16]:

(7) $$\begin{array}{c} \bar{F_{z,i}}=\Sigma P_{i,j}\left(\xi_{j+1}^{\prime }-\xi_{j}^{\prime }\right), \end{array}$$

where *i* is the respective beam under consideration and $\xi_{j}^{\prime }$ and $\xi_{j+1}^{\prime }$ are the nodes on the cross-section as shown in Figure 2.

### 2.3. Hydrodynamic Coupling in Arrays

We focus in this section on the hydrodynamic coupling effects between members in an array. We provide a generalized matrix formulation for an array of beams incorporating coupling contributions of all members to study the influence of non-neighbouring members in a three- and a five-beam array.

The derivations of the hydrodynamic coupling matrix of the nearest neighbour model are based on existing work by Basak and Raman [16]. Our new contribution with this work is the consideration of all members, analysis and discussion of coupling for different gap widths and Reynolds numbers in comparison to only nearest neighbour influence. We consider identical beams (geometrical and material properties) equally spaced apart. The width of each beam is 2b and the gap between members is 2g, see Figure 1. The unsteady stream function in (6) is computed for transverse vibrations and the velocity matching conditions are formulated for *M* beams. The transverse velocity of the $m^{th}$ beam in the array is given by $W_{m}\left(z\right)={\hat{W}}_{m}e^{i(\omega t+\theta_{m})}$, where ${\hat{W}}_{m}$ is the velocity amplitude and $\theta_{m}$ is the phase of vibration of the $m^{th}$ beam. A system of uncoupled integral equations is found by substituting the expression for streamfunction in boundary conditions (Equation (3)) to solve for unknown vorticities and pressures. We nondimensionalize gaps, pressure jumps and velocity amplitudes to make comparisons meaningful. The uncoupled integral equations are then solved using the Gauss–Legendre quadrature method. The hydrodynamic matrix elements for an array of *M* beams incorporating interactions between all members in the array are given by

$$G_{mn}={\left[\begin{array}{c} A_{kj} \end{array}\right]}_{mn},$$

where m,n∈1…M and $A_{kj}$ is given by

(8) $$\begin{array}{cc} A_{kj} & =\int_{\xi_{j}}^{\xi_{j+1}}L(\sqrt{iRe}|\xi^{\prime }-\xi \left|\right)d\xi^{\prime },\\ \; & =\frac{1}{2\pi }\left[f\left(Re,\xi_{j+1}^{\prime },\xi_{k}\right)-f\left(Re,\xi_{j}^{\prime },\xi_{k}\right)\right], \end{array}$$

where the kernel function

$$L=\Psi_{\xi \xi^{\prime }}$$

is a particular Green’s function given by

(9) $$\Psi =-\frac{1}{2\pi \alpha^{2}}\left(lnR+K_{0}\left(\alpha R\right)\right)$$

where $\alpha =\sqrt{iRe}$, $K_{0}$ is the modified Bessel function of third kind, order zero and $R=\sqrt{{\left(y-y^{\prime }\right)}^{2}+{\left(z-z^{\prime }\right)}^{2}}$ and

(10) $$\begin{array}{cc} f(Re,{\xi_{j}}^{\prime },\xi_{k}) & =\frac{i}{Re}\left(\frac{1}{\xi_{j}^{\prime }-\xi_{k}}+sgn\left(\xi_{j}^{\prime }-\xi_{k}\right)i\sqrt{iRe}K_{0}\left(-i\right|\xi_{j}^{\prime }-\xi_{k}\left|\sqrt{iRe}\right)\right). \end{array}$$

where $\xi_{j}^{\prime }$ is any node on the beam, $\xi_{k}$ is the midpoint between any two nodes, see Figure 2.

Each diagonal entry in the coupling matrix $\hat{G}$ represents the hydrodynamic influence coefficients of segments of the same beam with itself, whereas each off-diagonal entry comprises elements representing the hydrodynamic coupling generated by the neighbouring ((m+1)th and (m−1)th) and non-neighbouring ((m+i) th and (m−i)th) (where *i* is not equal to 1) members on the *m*th beam. For example, $G_{11}$ contains hydrodynamic influence coefficients on the first beam from segments of the same beam, whereas $G_{12}$ contains hydrodynamic influence coefficient of the first beam subject to the influence of the segments of the second beam, and so on. In order to compute the elements of sub-matrix $G_{11}$ a loop is run over the number of nodes *j*(0,...,N) for each midpoint *k*(0,...,N−1).

The matrix-vector equations can be written as follows

(11) $$\begin{array}{c} {\left[1\;0...0\right]}^{T}=\hat{G}{\left[P_{m}\right]}^{T}, \end{array}$$

where the amplitudes and phases of members in the array are relative to beam 1. We consider beam 1 as our actuated beam.

The coupling matrix $\hat{G}$ incorporating all member interactions for a three and a five-beam array are given by:

$${\hat{G}}_{III\;}=\;\left[\begin{array}{ccc} G_{11} & G_{12} & G_{13}\\ G_{21} & G_{22} & G_{13}\\ G_{31} & G_{32} & G_{33} \end{array}\right],$$

$${\hat{G}}_{v\;}=\;\left[\begin{array}{ccccc} G_{11} & G_{12} & G_{13} & G_{14} & G_{15}\\ G_{21} & G_{22} & G_{23} & G_{24} & G_{25}\\ G_{31} & G_{32} & G_{33} & G_{34} & G_{35}\\ G_{41} & G_{42} & G_{43} & G_{44} & G_{45}\\ G_{51} & G_{52} & G_{53} & G_{54} & G_{55} \end{array}\right],$$

where the elements in red represent the coupling contributions of non-neighbouring members and are zero in existing literature by Intartaglia et al. [12], Basak and Raman [16], Tung et al. [23]. However, here we do not set them to zero. The nondimensional pressure jumps are found simply by multiplying (11) by the inverse of coupling matrix $\hat{G}$.

## 3. Hydrodynamic Loading

In what follows we investigate the hydrodynamic loading acting on thin beams subject to small transverse oscillations in an unbounded fluid domain. We solve the hydrodynamic loading for varying gap widths between members and for different Reynolds numbers. First, we validate results for a single beam and a pair of beams with existing results in the literature and then analyse the hydrodynamic loading on a three- and a five-member array.

### 3.1. Validation

**Figure 3:**
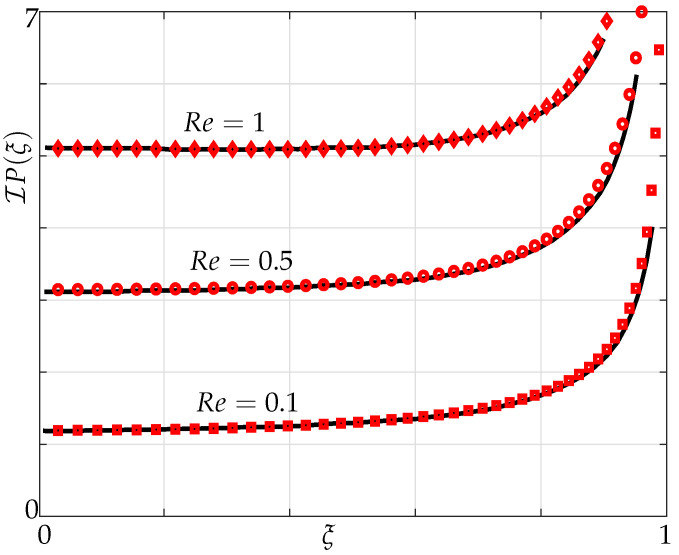
Variation of the imaginary part of pressure difference across the beam for Re=[0.1,0.5,1]; our simulations Equation (5) (markers) and Tuck’s results (solid lines).

**Figure 4:**
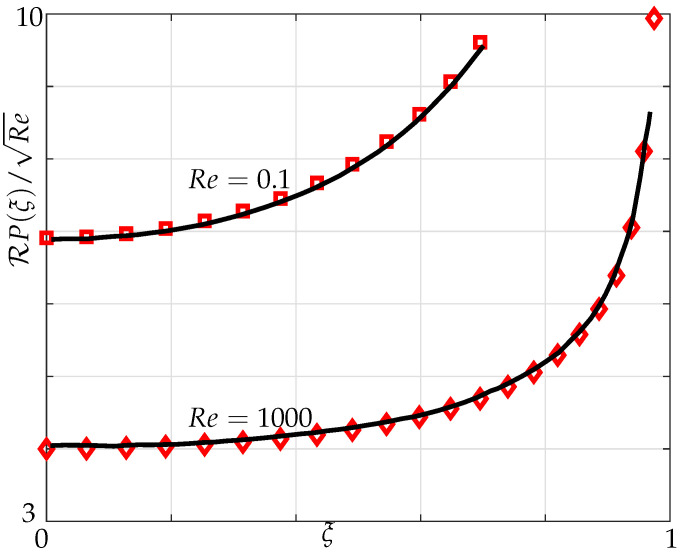
Variation of the real part of pressure difference across the beam for Re=[0.1,1000] scaled with respect to square root of the Reynolds number; our simulations, Equation (5) (markers), and Tuck’s results (solid lines).

The pressure differences between the top and bottom surfaces across the width of an infinitely thin beam are shown in the Figure 3 and Figure 4 with the imaginary component of the pressure denoting the added mass and the real component of the pressure denoting the dissipative effects [20].

We first validate for hydrodynamic loading acting on a single beam of finite width and negligible thickness undergoing small transverse vibrations in an unbounded fluid domain against results obtained by Tuck [20] in which he restricted it to flows caused by movement of cylindrical bodies.

In Figure 3 and Figure 4, variation of the imaginary and real parts of pressure are plotted for different Reynolds numbers. We note from Figure 3 that the pressure curve starts to become concave down towards the centre, i.e., ξ=0 with increasing Reynolds numbers. We observe a good agreement in both imaginary and real parts of pressure curvatures at different Reynolds numbers with Tuck’s results.

**Figure 5:**
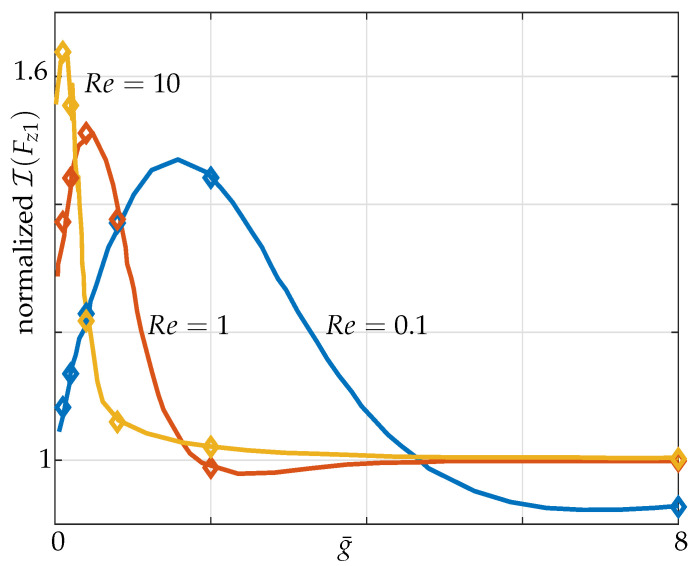
Variation of the imaginary part of nondimensional hydrodynamic force across microbeam 1 for two beams vibrating in-phase (1–1 configuration) over $\bar{g}$ normalized by their corresponding values at the same Re in an unbounded fluid with solid lines representing Basak’s results and markers our validation against it.

Next, we consider a two-beam configuration in which both beams are actuated at their maximum amplitudes, i.e., a 1–1 configuration. The nondimensional transverse force per unit length on microbeam 1, $F_{z1}$ as a function of $\bar{g}$ is plotted for different Re values (see Figure 5) and is validated against the results provided by Basak and Raman [16].

### 3.2. Hydrodynamic Coupling of a Pair of Beams

Basak and Raman [16] considered the beams to be hydrodynamically decoupled when the hydrodynamic loading over each beam resembles that on an isolated beam. For validation purposes, we considered Basak’s 1–1 actuation scenario where 1 denotes an actuated beam. Note that our investigations, however, emphasize the 1–0 case in which 0 denotes the passive beam. We do so to investigate the interactive coupling effects as opposed to the hydrodynamic coupling (1–1) considered by Basak.

**Figure 6:**
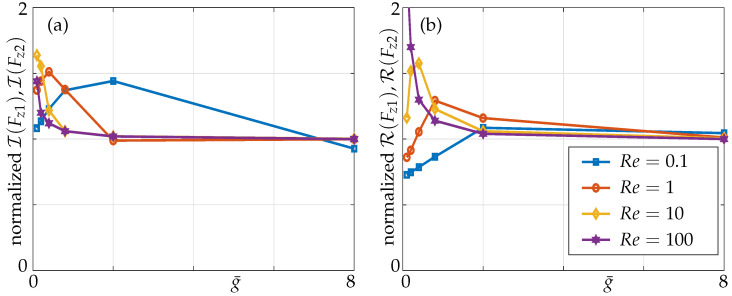
Hydrodynamic load of 2 beams for different gap sizes $\bar{g}$ subject to a 1–1 actuation mode; (**a**) imaginary parts of forces, (**b**) real parts of forces; note that lines of beams 1 and 2 perfectly align with the solid lines representing Basak’s results and marks our validation against it.

**Figure 7:**
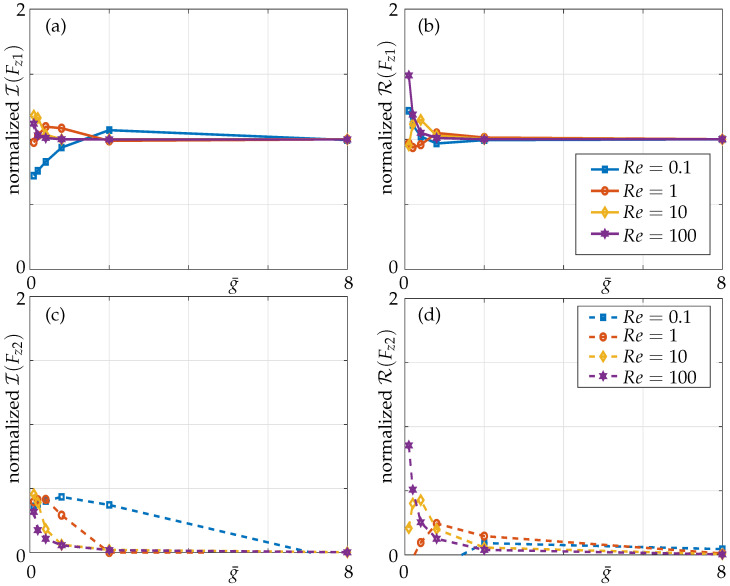
Hydrodynamic load of 2 beams for different gap sizes $\bar{g}$ subject to the 1–0 actuation mode; (**a**,**c**) imaginary parts of forces, (**b**,**d**) real parts of forces; (**a**,**b**) actuated beam 1, (**c**,**d**) passive beam 2.

In Figure 6, the nondimensional transverse force per unit length (Equation (7)) on beams 1 and 2 as a function of $\bar{g}$ is plotted for different Re values and is validated against results provided by Basak and Raman ([16], Figure 7). Pressure differences for larger Re results in a smaller boundary layer thickness and it scales as 1/$\sqrt{Re}$, implying that the pressure and velocity fields are more localized, resulting in weak interactions between beams in the array. However, for smaller Re, boundary layer thickness is larger, resulting in overlapping boundary layers between the beams for a given gap. A significant difference can be noticed from Figure 6 as the hydrodynamic loading approaches its corresponding isolated beam value at a much larger gap $\bar{g}$ at Re=0.1 compared to a smaller gap at Re=100 [16].

Here we study the interactive coupling effects, unlike Basak and Raman who explored the 1–1 configuration in which both beams were oscillating at their maximum amplitudes. Though Basak and Raman have defined two beams to be hydrodynamically decoupled when the hydrodynamic loading on each beam reaches 99% of its corresponding value of an isolated beam vibrating in an unbounded fluid domain, the relative effects of beam 2 with respect to beam 1 do not give any meaningful information about the mutual coupling. For instance, for any gap width between the beams, the ratio of hydrodynamic loading on beam 2 with respect to beam 1 is always the same for this particular configuration in which both beams are actuated and oscillating in-phase. However, in applications such as AFM, where each member of an array is required to portray exact measurements of the tip-sample scenario, the slightest interactive coupling from a neighbour member or any member in an array would cause a false-positive of that measurement. Hence, in our work we emphasize the mutual coupling and use the term “interactive coupling”, which is defined as the relative influence of the passive beam with respect to the active beam. Therefore, we excite one beam and keep remaining beams passive and the results for the hydrodynamic forces are shown in Figure 7. Moreover, another study by our group explores the shifting of stimulus, i.e., the actuated beam in the array and its effects on overall hydrodynamic load in small-sized arrays for varying gaps and Reynolds numbers [24].

## 4. Results

Nondimensional parameters that influence the coupled hydrodynamics are the gap $\bar{g}=g/b$, the amplitude ratio $r_{m1}$, the relative phase $\theta_{m1}$, the Reynolds number Re and the non-neighbouring members. Here only the left-most beam is actuated while the actuation of interior beams and other actuation configurations have been addressed in another article by the same authors [24].

### 4.1. Three Beam Array

The influence of the gaps between the beams and the effect of non-neighbouring members on the overall array dynamics are analyzed by comparing the absolute, imaginary and real values of hydrodynamic loading over the beams for different Reynolds numbers.

#### 4.1.1. Effect of the Gap

**Figure 8:**
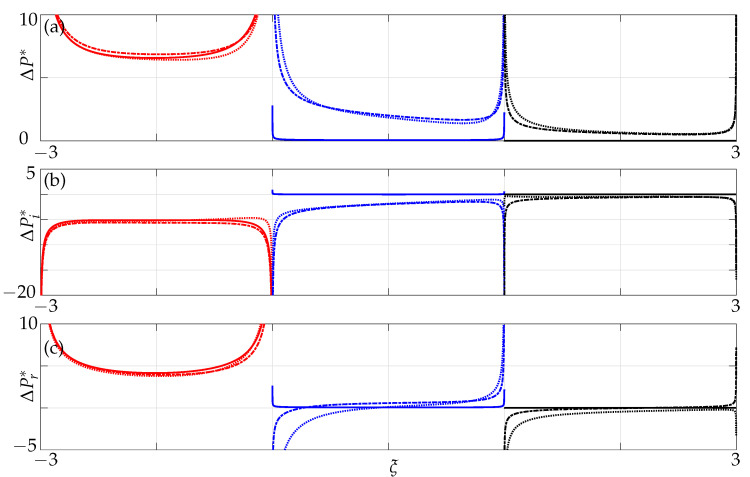
Variation of the (**a**) absolute, (**b**) imaginary and (**c**) real values of nondimensional pressure difference across three beams incorporating only the nearest neighbour influence at Re=1; solid lines: $\bar{g}=8$, dash–dotted lines: $\bar{g}=0.4$, dotted lines: $\bar{g}=0.1$, with the left-most beam active and the rest passive.

The absolute, imaginary and real parts of nondimensional pressure differences across the three beams with only nearest neighbour influence are plotted in Figure 8, for different nondimensional gaps $\bar{g}=g/b$ between the beams at Re=1.

We observe that, when the beams are far apart (solid lines) in the case of the only nearest neighbours considered, i.e., for a gap of $\bar{g}>8$, beams 2 and 3 are hydrodynamically decoupled from the actuated beam 1. Furthermore, the pressure difference across the width of the beams is symmetrical for larger gaps, see Figure 8. However, as the gaps between the beams decrease, the passive beams become hydrodynamically coupled, with beam 2 being significantly influenced (28%) in comparison to beam 3 (10%) for $\bar{g}=0.1$, implying that the coupling strength decreases with increasing distances from the active beam as observed before [16]. Note that the percentage of coupling influence is calculated by taking ratios of the pressure differences at the midpoints of the passive beam with respect to the active beam. The pressure differences across the width of the beams become asymmetric as the gaps decrease indicating a shift from a configuration of individual oscillators to that of an array. The pressure profile over the entire array is established by the position of energy input (beam 1 here) and load distributed according to gap width and Reynolds number. It is our strong hypothesis at this stage (without being able to provide an in-depth analysis or proof) that the onset of array coupling is strongly accompanied with/by inducing array vibration modes in the classical sense for structures. 

**Figure 9:**
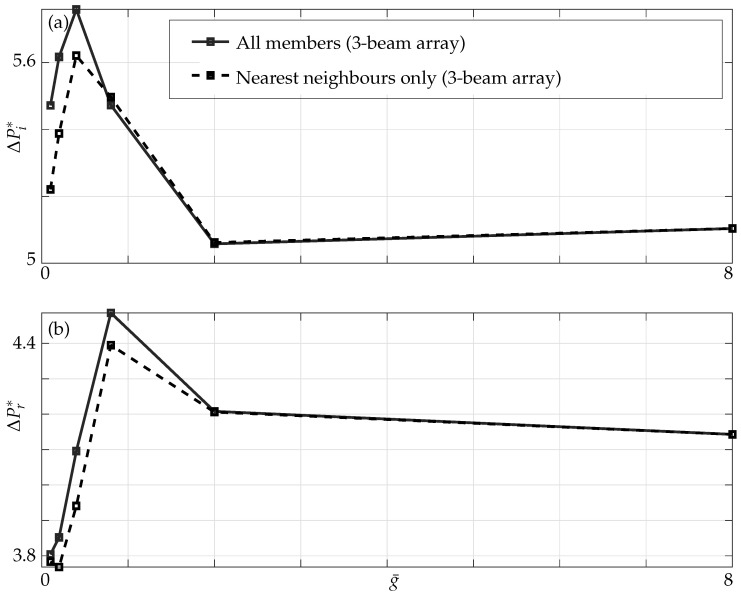
(**a**) Imaginary and (**b**) real parts of nondimensional pressure evaluated at the mid point of the active beam in a three-beam array (1–0–0 configuration) for different nondimensional gaps; nearest neighbours only (dashed lines) and all members incorporated (solid lines) at Re=1.

In Figure 9, we observe that, as the gaps between the beams decrease, the added mass and damping effects evaluated at the midpoint of each beam behave in non-intuitive ways. In addition, the range of $\bar{g}$ over which added mass and damping effects display their maximum is different, for instance the critical gaps for added mass is $\bar{g}=0.4$ (a difference of ≈3% is seen when all members are incorporated) while for damping it is $\bar{g}=0.8$ which agrees with the existing results in the literature, resulting in constructive or destructive zones of hydrodynamic interference [16].

#### 4.1.2. Effect of the Non-Neighbouring Members

**Figure 10:**
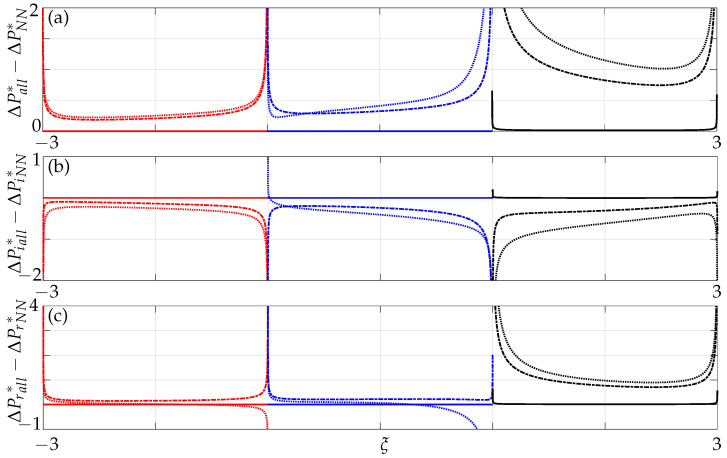
Difference in (**a**) absolute, (**b**) imaginary and (**c**) real values of nondimensional pressure difference across three beams incorporating all members and when only nearest neighbours are incorporated at Re=1; solid lines: $\bar{g}=8$, dash-dotted lines: $\bar{g}=0.4$, dotted lines: $\bar{g}=0.1$, with the left-most beam active and the rest passive.

Next, the differences in nondimensional pressure are plotted when all members are incorporated as opposed to when only nearest neighbour effects are incorporated. For a gap $\bar{g}>8$, the pressure difference represented by the solid line is zero, implying that non-neighbouring members do not affect the array dynamics and, hence, can be neglected for larger gaps as each beam behaves as a single beam vibrating in an unbounded fluid domain, see Figure 10. As the gap decreases between the beams, the significance of non-neighbouring beams’ contribution to the overall dynamic behaviour increases with decreasing gaps. Moreover, unlike in the case with only nearest neighbours we note here an increase in coupling influence further away from the active member (see beam 3 in Figure 10). This implies that there is a transfer of energy not only between nearest neighbours but between non-neighbours as well, especially significant for smaller gaps, i.e., $\bar{g}<0.4$. The pressure difference on beam 3 (0.82) at a critical gap of $\bar{g}=0.4$ is comparatively higher than that of beam 2 (0.32), implying that the non-neighbouring beams do play a significant role. This further strengthens our hypothesis stated earlier, that array modes become influential at a critical gap size.

We now investigate the significance of non-neighbouring members by plotting the differences in imaginary and real parts of pressure when all members are incorporated and compare it to the results when only nearest neighbours are incorporated. From Figure 10 we observe that the added mass and damping computed at the midpoint of beam 3 is higher in comparison to beam 2. Hence, beam 3 is significantly influenced when non-neighbouring beams are incorporated. When the effect of non-neighbouring beams are ignored the beam closest to the active beam is significantly coupled and the coupling strength decreases with decreasing distances from the active beam. However, non-intuitive behaviour on beam 3 signifies that non-neighbouring beams do play a significant role and hence, cannot be ignored especially with decreasing gaps as they affect the overall array dynamics. Note (on beam 1), that there is also a difference on beam 1, not just on beams 2 and 3 which will have implications in AFM technology comprising of three beams in which one cannot neglect the influence of non-neighbouring member.

#### 4.1.3. Effect of the Reynolds Numbers

We also present the coupling ratios, i.e., the ratio of absolute values of nondimensional hydrodynamic force (7) of passive beam ($\bar{F_{p}}$) to that of the active beam ($\bar{F_{a}}$) for different Reynolds numbers, whereas $F_{21}$ and $F_{31}$ denote the ratio of hydrodynamic forces of the passive beams (2 and 3, respectively) with respect to the active beam 1.

**Figure 11:**
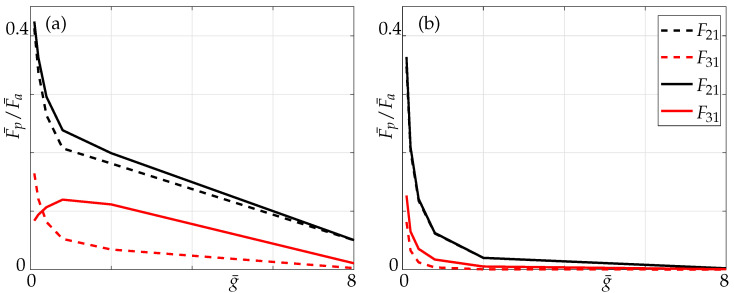
Coupling ratios of the passive beam with respect to active beam over a range of gaps in a three-beam array for the 1–0–0 configuration at (**a**) Re = 0.1 and (**b**) Re = 100; nearest neighbours only (dashed lines) and all members (solid lines).

**Figure 12:**
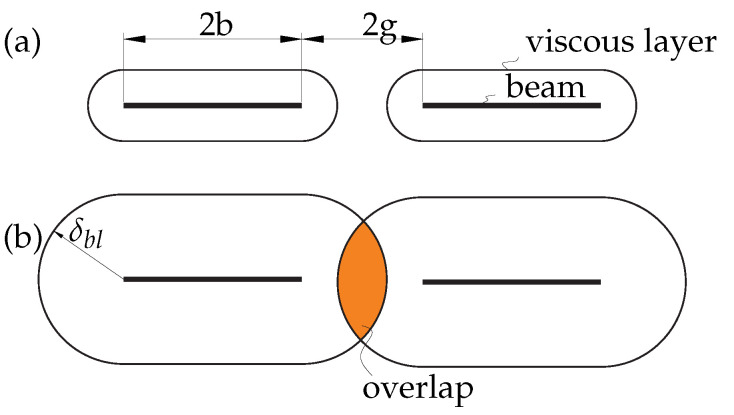
Localized boundary layers for higher Reynolds number (**a**) Re=100 and (**b**) overlapping boundary layers for lower Reynolds number Re=1.

In Figure 11 we observe that for lower Reynolds number, i.e., Re=0.1 in this instance, there is a noticeable difference on the impact of non-neighbouring members. This can be explained by the fact that the boundary layers overlap (see Figure 12) whereas for higher Reynolds numbers, i.e., Re=100 the influence of non-neighbouring members can be neglected due to inviscid flow limit.

### 4.2. Five Beam Array

A similar structure is followed, as in the case of a three-beam array in which we study the influence of the gaps between the beams and the effect of non-neighbouring members on the overall array dynamics by comparing the absolute, imaginary and real values for different Reynolds numbers.

#### 4.2.1. Effect of the Gap

**Figure 13:**
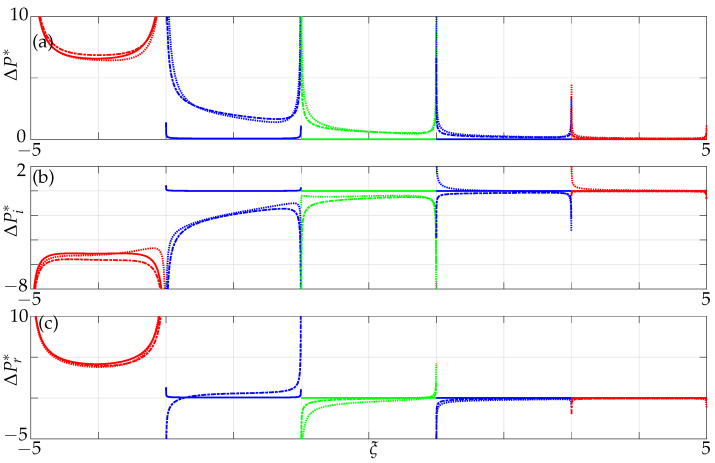
Variation of the (**a**) absolute, (**b**) imaginary and (**c**) real values of nondimensional pressure difference across five beams incorporating only nearest neighbour influence at Re=1; solid lines: $\bar{g}=8$, dash-dotted lines: $\bar{g}=0.4$, dotted lines: $\bar{g}=0.1$, with the left-most beam active and the rest passive.

The imaginary and real parts of nondimensional pressure difference across the five beams with only nearest neighbour influence are plotted in Figure 13 for different nondimensional gaps $\bar{g}$ between the beams for Re=1.

We observe again that when the beams are far apart, i.e., for a gap of $\bar{g}=8$, all passive beams are hydrodynamically decoupled from beam 1 (beam 1 is moving while beams 2–5 are stationary). As the distance from the active beam increases, the hydrodynamic coupling strength decreases for the passive beam under consideration. Furthermore, we notice a similar trend in pressure difference distribution for larger gaps with it being symmetrical, and for increasing influence the nondimensional pressure jump becomes distorted and asymmetrical.

**Figure 14:**
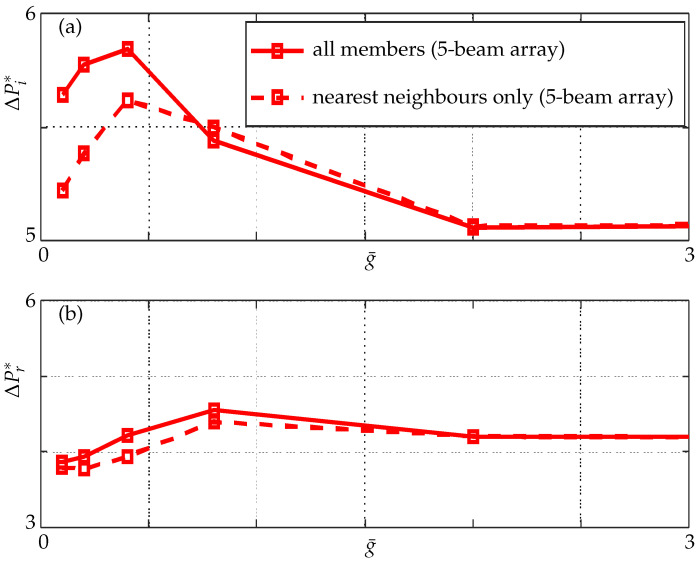
(**a**) Imaginary and (**b**) real parts of nondimensional pressure evaluated at the mid point of the active beam in a five-beam array (1–0–0–0–0 configuration) for different nondimensional gaps; nearest neighbours only (dashed lines) and all members incorporated (solid lines).

The added mass and damping display a non-monotonic trend here as observed in a three-beam array with the magnitude of added mass and damping (compare Figure 9 and Figure 14) being different to that in a three-beam array at the same critical gaps.

#### 4.2.2. Effect of the Non-Neighbouring Members

We consider the influence of all members and plot the differences in absolute, imaginary and real parts of nondimensional pressure when all beams and when only nearest neighbours are incorporated to study the influence of non-neighbouring members in a five-beam array at a particular Reynolds number Re=1.

**Figure 15:**
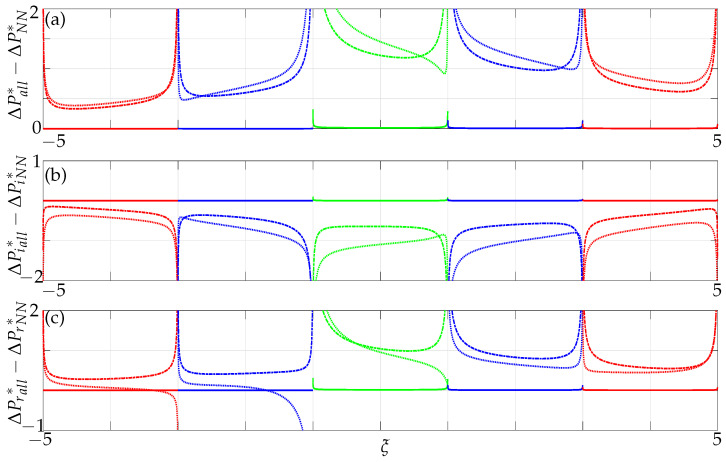
Difference in (**a**) absolute, (**b**) imaginary and (**c**) real values of nondimensional pressure difference across five beams incorporating all members and when only nearest neighbours are incorporated at Re=1; solid lines: $\bar{g}=8$, dash-dotted lines: $\bar{g}=0.4$, dotted lines: $\bar{g}=0.1$, with the left-most beam active and the rest passive.

A similar effect is observed as in the case of a three-beam array for a nondimensional gap $\bar{g}>8$ implying that non-neighbouring members can be neglected for such gaps as they do not affect the overall array dynamics, see Figure 15. However, they increasingly become significant with decreasing gaps between the beams and with increasing array size. From Figure 15 we note that the active beam (beam 1) is also affected when non-neighbouring members are incorporated and not just the passive beams, i.e., (beam 2, beam 3, beam 4 and beam 5), further strengthening our argument from the case of a three-beam array of the onset of array effects with increasing size of the array and decreasing gaps. This implies that one cannot ignore the collective dynamics with increasing size of the array and in particular for decreasing gaps. 

#### 4.2.3. Effect of the Reynolds Numbers

**Figure 16:**
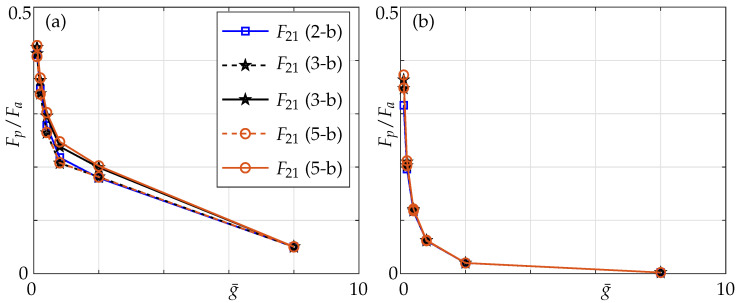
Coupling ratio of the passive to the active beam for different configurations for (**a**) Re=0.1; (**b**) Re=100; nearest neighbours: dashed line; all members: solid lines; with 1–0: blue, 1–0–0: black and 1–0–0–0–0: orange denoting the respective configurations under consideration.

In this section we study the effects of Reynolds numbers and make comparisons against coupling effects in a three-beam array. As can be seen from Figure 16, at Re=0.1, the coupling ratio is slightly higher, especially with decreasing gaps and increasing array size. Comparing the coupling ratios in a three- and a five-beam array to that of multiple beams, we observe that when only nearest neighbours are incorporated the coupling ratio in the arrays is lower than the multiple beam case, while it is higher when all members are incorporated.

At Reynolds number 100, coupling effects are independent of the non-neighbouring member effects and size of the array. This is because the boundary layers are more localized and, hence, when non-neighbouring members are incorporated one is still not able to distinguish between a 1–0–0 and a 1–0–0–0–0 configuration. This implies that at higher Reynolds numbers, neither the number of members in the array nor the effect of non-neighbouring members matter due to shrinking boundary layers (see Section 3) but a slight difference can be noticed for very small gaps.

Furthermore, a sharp increase in coupling can be observed for $\bar{g}<8$ at Re=0.1, whereas it can only be seen for $\bar{g}<2$ at Re=100, see Figure 16. This can again be attributed to the overlapping boundary layers (see Figure 12) at much larger distances for low Re compared to it overlapping for smaller gaps at high Re. With increasing number of members in the array, a significant difference in the mode shape can be observed (compare the absolute parts of pressure in Figure 10 and Figure 15). The mode shape resembles a symmetric downward facing parabola in a five-beam array compared to it resembling an increasingly convex shaped mode in a three-beam array justifying our hypothesis stated earlier with respect to onset of array effects.

## 5. Conclusions

The underlying physics of the hydrodynamic interactions between multiple members in a three- and a five-beam array have been systematically analyzed for active–passive configuration with and without the incorporation of non-neighbouring members for different Reynolds numbers and different gap widths between members. We incorporated additional coupling contributions due to non-neighbouring members to investigate their effects of added mass and damping on the whole array (Section 2.3).

Non-neighbouring members are insignificant for higher Re irrespective of the gap width between the beams due to the localized effects, i.e., shrinking boundary layers. However, for lower Re one cannot neglect the effects of non-neighbouring members due to the overlapping boundary layers or global effects but can be ignored for large gaps, i.e., $\bar{g}>2$. In addition, the overall hydrodynamic response demonstrates a different behaviour, i.e., has different orders of magnitude at lower and higher Reynolds numbers.

In terms of specific applications, i.e., building blocks, one must expect the influence of non-neighbouring blocks for gaps less than the critical gap, i.e., ${\bar{g}}_{critical}<2$ to minimize damage to the structure in the event of a heavy gust. For instance, in New York, USA the blocks of buildings are densely packed with gap sizes between buildings of $\bar{g}<1$ and, hence, it is important to take into account the effect of non-neighbouring blocks.

**Figure 17:**
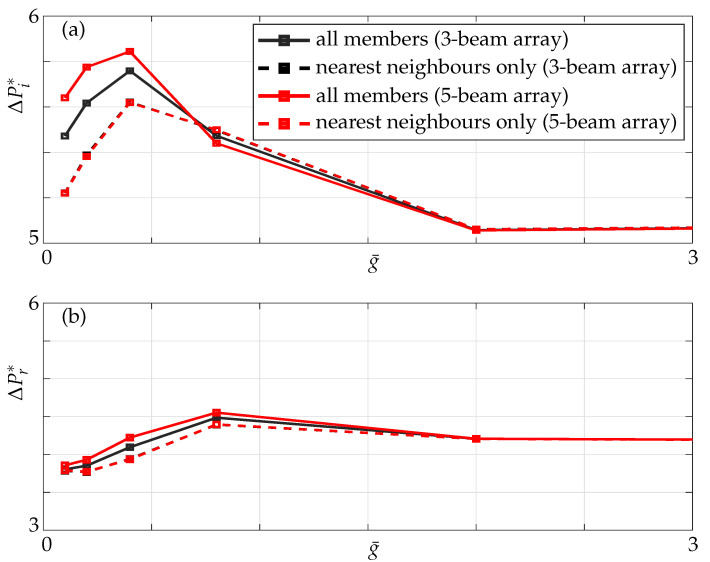
(**a**) Imaginary and (**b**) Real parts of pressure at the mid point of active beam in a three- (black) and a five-beam (red) array for different gaps with only nearest neighbours (dashed line) and all members incorporated (solid line) at Re=1.

Non-neighbouring members strongly impact the added mass and damping for $\bar{g}<2$ and, in particular, the effect is enhanced for $\bar{g}<0.4$ implying that they cannot be ignored, see Figure 17. At the micro-scale, in an AFM array with increasing number of cantilevers, one cannot ignore the influence of non-neighbouring members, i.e., if beam 1 is sensing the sample it is critical to take into account the effects of beam 3, in a three-beam array. In the instance of a four-cantilever AFM array, cantilever 3 could have picked up the dust particle sensed by cantilever 2, but in reality it is an artifact. Such artifacts may arise from the fact that the effect of non-neighbouring member was not considered when designing a 4-beam array with typical gap size $\bar{g}\approx 0.1$ between members. It becomes even more critical with more beams.

Both added mass and damping effects significantly impact the collective array dynamics, especially with increased array size. A key conclusion is that non-neighbouring members play a significant role as the size of the array increases and cannot be ignored for arrays with more than three members. A conservative measure is that large arrays can be treated as a series of individual oscillators given the gap between each oscillator is sufficiently large, i.e., $\bar{g}>8$. However when the oscillators are in close proximity to each other, i.e., $\bar{g}<2$, additional effects are observed due to the significance of non-neighbouring members implying that array effects dominate the overall dynamics.

## Data Availability

The data that support the findings of this study are available from the corresponding author, A.K.M., upon reasonable request.

## Abbreviations

The following abbreviations are used in this manuscript:

MEMS	Micro Electro Mechanical Systems
IPMCs	Ionic Polymer Metal Composites
AFM	Atomic Force Microscope
